# UHPLC‐QTOFMS Urine Drug Screening With Dilute‐and‐Shoot Sample Preparation and Vacuum‐Insulated Probe‐Heated Electrospray Ionization

**DOI:** 10.1002/dta.3830

**Published:** 2024-11-12

**Authors:** Mira Sundström, Pirkko Kriikku, Ilkka Ojanperä, Carsten Baessmann, Anna Pelander

**Affiliations:** ^1^ Forensic Chemistry Unit Finnish Institute for Health and Welfare (THL) Helsinki Finland; ^2^ Department of Forensic Medicine University of Helsinki Helsinki Finland; ^3^ Bruker Daltonics Bremen Germany

**Keywords:** dilute‐and‐shoot, high‐resolution mass spectrometry, time‐of‐flight mass spectrometry, urine drug screening, vacuum‐insulated probe‐heated electrospray ionization (VIP‐HESI)

## Abstract

We developed a method for comprehensive urine drug screening by applying dilute‐and‐shoot extraction and vacuum‐insulated probe‐heated electrospray ionization with ultra‐high performance liquid chromatography high‐resolution quadrupole time‐of‐flight mass spectrometry (DS‐UHPLC‐VIP‐HESI‐QTOFMS). The method involved five‐fold post‐hydrolysis dilution of urine samples and chromatography on a C18 UHPLC column prior to QTOFMS analysis. The recently introduced VIP‐HESI ion source was chosen due to its enhanced ionization efficiency and compatibility with UHPLC‐QTOFMS. Extensive data was acquired in positive ion mode with a low collision energy (7 eV) and an elevated collision energy (30 eV), using the broadband collision‐induced dissociation data acquisition scan mode that continuously generated high‐resolution and accurate mass for parent and fragment qualifier ions, and parent ion isotopic patterns. Compound identification was performed against an in‐house database with 1263 compound entries, using an automated post‐run reverse target database search with preset identification criteria. Method validation with 56 different drugs showed acceptable results for the limit of identification (median 5 ng/mL), matrix effects (70–130%), repeatability of retention times (< 1%), mass accuracy (< 1 mDa), as well as for specificity and stability. As compared with an established UHPLC‐QTOFMS method relying on solid‐phase extraction and conventional electrospray ionization, DS‐UHPLC‐VIP‐HESI‐QTOFMS produced comparable results from authentic clinical urine samples for most drugs, but showed clearly improved detectability for pregabalin, gabapentin, and ritalinic acid. We anticipate that the new method will be a step forward for laboratories performing routine urine drug screening due to its fast turnaround time, reduced manual workload, cost efficiency, and broad substance coverage.

## Graphical abstract

The new ionization technique, vacuum‐insulated probe‐heated electrospray, allows for a simple dilute‐and‐shoot sample preparation to be used with liquid chromatography time‐of‐flight mass spectrometry in urine drug screening. Comparison with a method relying on solid‐phase extraction and conventional electrospray ionization shows improved performance with amphoteric hydrophilic drugs such as pregabalin.

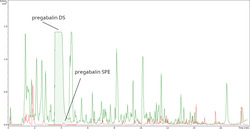

## 1 Introduction

There has been constant evolution in liquid chromatography time‐of‐flight mass spectrometry (LC‐TOFMS) drug screening methods over the more than 20 years that TOFMS has been available to analytical toxicologists in the form of bench‐top instruments [1]. Our laboratory was among the first to introduce accurate mass‐based TOFMS with full‐spectrum acquisition to urine drug screening, first as a proof of concept using single TOFMS instrumentation [2, 3], and later, along with the improvement of mass accuracy and resolving power, elaborating the concept into a stand‐alone toxicology laboratory tool using quadrupole TOFMS (QTOFMS) [4].

These methods have regularly relied on solid‐phase extraction (SPE) in our laboratory and in many other laboratories, offering a compromise between scope, sensitivity, and extract purity. However, time‐consuming sample preparation using SPE is not an optimal choice in an operating environment where minimal sample turnaround time is necessary. Generic sample preparation using the dilute‐and‐shoot (DS) approach is an attractive option for comprehensive drug screening in terms of scope, simplicity, high throughput, cost efficiency, and lower solvent and sample volume requirements. The main purpose of sample dilution is to reduce matrix interferences, thus allowing for better sensitivity [5, 6, 7].

Until now, DS has generally been used in connection with triple quadrupole MS (QqQ) operated in selected reaction monitoring mode. After the first LC‐QqQ‐based direct urine drug analysis methods in the early 2000s [8, 9, 10], many other DS studies have been published [11, 12, 13, 14, 15, 16, 17, 18, 19, 20, 21, 22, 23, 24]. However, with the increase in the number of drugs to be monitored, including new psychoactive substances (NPS), the pre‐targeted LC‐QqQ methods begin to suffer from a lack of sensitivity due to a reduced number of data points per peak. The number of analytes monitored in a single analytical run with LC‐QqQ can remain limited, sometimes requiring two consecutive analyses [22]. A broader scope can be obtained by applying high‐resolution mass spectrometry (HRMS), either using QTOFMS or Orbitrap technology, with non‐targeted data acquisition. HRMS enables high mass accuracy and high mass resolving power without the need for analyte preselection.

Comprehensive drug screening methods involving DS and HRMS are still scarce in analytical toxicology. Here, our objective is to demonstrate the potential of vacuum‐insulated probe‐heated electrospray ionization (VIP‐HESI) combined with a simple DS method for urine drug screening by ultra‐high performance liquid chromatography (UHPLC) and QTOFMS. Despite the advantages offered by HRMS, the DS extract obtained from urine is very complex and thus places high demands on the mass analyzer's performance and data processing. In order to achieve sufficient sensitivity while minimizing matrix interference, we ended up using the recently developed ionization technology, VIP‐HESI.

This new ion source has some features that help improve the performance of the DS‐UHPLC‐VIP‐HESI‐QTOFMS workflow. In general, heat addition improves the desolvation process and thus increases ion beam brightness and sensitivity. A vacuum layer between the electrospray ionization (ESI) probe heater and the eluent reduces heat transfer from the hot ceramic heater to the sample. This allows heat addition after the nebulizer and protects thermally labile compounds from thermal decomposition. Furthermore, active exhaust causes a pressure difference between the source chamber and the exhaust. This minimizes the recirculation of nebulized gases reducing memory effects and chemical noise while increasing the robustness of the ion source.

## 2 Experimental Section

### 2.1 Materials

Reference standards for drugs were purchased from various vendors and were of pharmaceutical purity. Water was purified using the Milli‐Q Integral 5 system equipped with a reverse‐phase silica cartridge LC‐PAK (Merck Millipore, Burlington, MA, USA). Reagents and solvents of analytical grade purity were from Honeywell (Charlotte, NC, USA), Merck (Darmstadt, Germany), and VWR International (Radnor, PA, USA). β‐glucuronidase was from Roche (Mannheim, Germany). For the validation experiments, 10 healthy male and female volunteers provided drug‐free urine with varying characteristics (pH 5.14–7.55 and specific gravity 1.003–1.023). Authentic urine samples were submitted to the laboratory for routine toxicological analysis and anonymized for the purposes of this study.

The validation experiments were performed for 56 compounds that represented various pharmacological categories and chemical structures (listed in Table 1). The compounds were spiked in artificial urine [25] at concentrations between 10 and 200 ng/mL. This 56‐compound quality control (QC) mixture was also used for one‐point calibration to transfer positive drug findings in analyzed authentic urine samples to the laboratory information management system. The validation was performed on model compounds, which has been shown to be sufficient for this kind of multi‐analyte methods [26].

**TABLE 1:** Validation results and quality control concentration for 56 model compounds.

				Repeatability of RT		Repeatability of mass accuracy		Autosampler stability after 5 days (% change)	Matrix effects (%)
Compound	QC concentration (ng/mL)	RT (min)	LOI (ng/mL)	Intra‐assay (RSD%)	Inter‐assay (RSD%)	Median intra‐assay (mDa)	Median inter‐assay (mDa)		
**Alpha‐PHP**	40	8.36	4	0.00	0.03	0.04	0.09	5	89
**Alpha‐PVP**	40	7.12	4	0.07	0.06	0.11	0.10	6	89
**Alprazolam**	20	11.32	5	0.04	0.04	0.29	0.44	3	107
**7‐Aminoclonazepam**	20	6.37	10	0.07	0.10	0.11	0.11	4	66
**Amitriptyline**	40	10.71	4	0.03	0.04	0.13	0.14	8	99
**Amphetamine**	200	4.53	37	3.08	3.04	0.05	0.07	8	117
**Atomoxetine**	40	10.12	10	0.04	0.04	0.08	0.18	4	84
**Benzoylecgonine**	100	6.20	10	0.08	0.10	0.14	0.12	10	82
**Buprenorphine**	20	9.74	1.6	0.04	0.05	0.12	0.12	6	720
**Bupropion**	20	8.01	10	0.06	0.06	0.21	0.16	3	67
**Citalopram**	40	8.98	4	0.00	0.03	0.12	0.12	2	81
**Clozapine**	40	9.26	4	0.04	0.05	0.13	0.14	6	145
**Cocaine**	20	6.78	5	0.06	0.07	0.08	0.07	7	159
**Codeine**	100	2.74	10	1.03	1.57	0.12	0.11	12	71
**Desmethylcitalopram**	40	9.04	4	0.05	0.05	0.19	0.22	1	73
**Doxepin**	40	9.36	10	0.04	0.05	0.11	0.14	6	80
**EDDP**	40	9.13	4	0.05	0.04	0.17	0.18	9	91
**Fentanyl**	20	8.68	1	0.00	0.03	0.08	0.20	7	90
**Fluoxetine**	40	10.84	10	0.07	0.05	0.15	0.16	5	103
**Haloperidol**	40	9.57	4	0.05	0.05	0.13	0.16	9	76
**Hydroxyalprazolam**	20	10.91	10	0.04	0.05	0.12	0.18	3	173
**Hydroxybupropion**	100	7.45	10	0.06	0.07	0.04	0.08	4	69
**Hydroxymidazolam**	20	10.34	2	0.00	0.03	0.18	0.18	0	92
**3‐Hydroxyphenazepam**	20	11.40	10	0.08	0.06	0.20	0.25	1	107
**9‐Hydroxyrisperidone**	10	7.64	1	0.06	0.06	0.03	0.07	4	81
**Hydroxyzine**	40	10.67	4	0.04	0.03	0.16	0.18	5	85
**Lamotrigine**	100	6.94	25	0.07	0.06	0.08	0.11	3	66
**Levomepromazine**	40	10.66	10	0.05	0.04	0.17	0.19	7	362
**Lorazepam**	40	11.21	10	0.07	0.06	0.23	0.23	7	107
**MDMA**	40	4.76	10	1.53	1.54	0.11	0.08	5	83
**Methadone**	40	10.63	10	0.04	0.04	0.11	0.14	4	99
**Methamphetamine**	200	4.71	20	1.90	1.87	0.08	0.08	8	80
**Methylphenidate**	40	6.96	10	0.07	0.08	0.06	0.12	5	257
**Mirtazapine**	40	7.04	4	0.05	0.05	0.12	0.12	10	164
**Morphine**	100	1.59	45	1.49	1.67	0.42	0.50	10	34
**Naloxone**	20	2.62	7.5	2.18	1.92	0.16	0.19	17	46
**Norbuprenorphine**	10	8.58	2.5	0.00	0.04	0.14	0.29	6	89
**Norclozapine**	40	9.15	4	0.04	0.04	0.15	0.17	5	92
**Nordiazepam**	40	11.95	4	0.06	0.05	0.16	0.17	‐2	93
**Nordoxepin**	40	9.62	4	0.00	0.04	0.10	0.14	5	82
**Nortriptyline**	40	10.85	4	0.03	0.05	0.14	0.21	4	91
**O‐Desmethyltramadol**	40	4.79	10	1.40	1.40	0.10	0.10	9	70
**Orphenadrine**	40	10.02	4	0.04	0.05	0.12	0.14	4	88
**Oxazepam**	40	11.22	10	0.06	0.04	0.19	0.19	7	103
**Oxycodone**	40	3.21	10	1.12	1.17	0.31	0.28	4	64
**Paroxetine**	40	10.11	10	0.05	0.05	0.07	0.24	5	78
**Propranolol**	40	8.88	4	0.06	0.05	0.05	0.14	4	88
**Quetiapine**	40	9.49	4	0.05	0.05	0.20	0.17	4	107
**Risperidone**	10	8.18	1	0.00	0.00	0.15	0.20	10	209
**Sertraline**	40	11.28	10	0.07	0.05	0.18	0.20	2	100
**Temazepam**	100	11.60	10	0.06	0.04	0.21	0.20	0	129
**THC‐COOH**	30	14.80	10	0.08	0.07	0.24	0.32	12	121
**Tramadol**	40	6.84	4	0.07	0.07	0.08	0.08	5	89
**Venlafaxine**	40	8.61	4	0.05	0.05	0.06	0.13	9	83
**Vortioxetine**	40	11.74	4	0.06	0.05	0.22	0.17	5	119
**Zolpidem**	40	7.60	4	0.06	0.06	0.07	0.13	5	86

Abbreviations: alpha‐PHP, alpha‐pyrrolidinohexiophenone; alpha‐PVP, alpha‐pyrrolidinovalerophenone; EDDP, 2‐ethylidene‐1,5‐dimethyl‐3,3‐diphenylpyrrolidine; LOI, limit of identification; MDMA, methylenedioxymethamphetamine; ME, matrix effects; RT, retention time; THC‐COOH, 11‐nor‐9‐carboxy‐Δ9‐tetrahydrocannabinol; QC, quality control.

### 2.2 Sample Preparation

Urine samples (100 μL) were hydrolyzed with 10 μL of β‐glucuronidase overnight at 37 °C with 2 mM ammonium acetate buffer (190 μL). After hydrolysis, 10 μL of a mixture of internal standards (codeine‐d_3_, MDMA‐d_5_, JWH‐018‐d_11_, and THCA‐d_3_, with final urine concentrations between 10 and 50 ng/mL) and 190 μL of 80% methanol/0.1% formic acid was added, the sample was vortexed, centrifuged, and 10 μL injected for analysis.

### 2.3 Instrumentation and Software

The instrumentation comprised a Bruker Daltonics (Bremen, Germany) Impact II QTOFMS equipped with a VIP‐HESI ion source, an exhaust outlet, and a six‐port valve. The QTOFMS was interfaced with an Agilent (Santa Clara, CA, USA) 1290 Infinity II UHPLC system. The UHPLC included a binary pump with a built‐in degasser, a high‐capacity multisampler, and a column thermostat. HyStar 6.2 and otofControl 6.3 software (Bruker Daltonics) were used for instrument control, while TASQ 2023 (Bruker Daltonics) was used for the creation of sample sequences, automated post‐run data processing, and review of the analysis results.

A Waters column (HSS T3, 150 × 2.1 mm, 1.8 μm) and an equivalent pre‐column (5 × 2.1 mm) were used for chromatographic separation at 60 °C with a 300 μL/min flow rate. The multisampler was kept at 10 °C. The mobile phases were 2 mM ammonium acetate in 0.1% formic acid (A) and methanol (B). The gradient consisted of 20% B (0–2 min), from 20% to 95% B (2–14 min), 95% B (14–17 min), and back to 20% B (17–18 min). The pre‐run equilibrium time was 2 min, and the total run time was approximately 22 min.

The mass spectrometer was operated in positive ion mode and acquired MS and broadband collision‐induced dissociation (bbCID) data at *m/z* 50 to 700, cycling between two collision energies (7 eV and 30 eV). The spectra rate was 1.43 Hz. The capillary voltage was 4000 V. The probe gas flow rate was 4 L/min (450 °C) and dry gas 8 L/min (200 °C). The nebulizer gas pressure was set at 2.5 bar. Nitrogen was used for all three gases. Compressed air was used for the exhaust drain port. Both post‐acquisition internal and external instrument mass scale calibration were performed with sodium formate clusters (1 mM sodium hydroxide in isopropanol/0.2% formic acid (1:1 *v/v*)) with exact masses between 90.9766 and 634.8760.

Compound identification was performed by an automated post‐run reverse database search with preset criteria for the mass accuracy of precursor (±3 mDa) and qualifier ion (±3 mDa), precursor ion peak height (5000 cnts), and retention time (RT, ±0.30 min). A lower precursor ion intensity threshold was applied for cannabinoids (3000 cnts), whereas a higher threshold (15,000 cnts) was used for gabapentinoids. Precursor ion isotopic pattern match was used as an additional parameter for compound identification. During this study, the size of the in‐house database was 1263 compound entries, for 980 of which reference material and thus full compound identification details were available (RT, precursor ion, isotopic pattern, and 0–2 assigned qualifier ions). In addition, the database included the exact masses for the glucuronides of buprenorphine, codeine, and morphine, the absence of which ensures the completion of enzymatic hydrolysis.

### 2.4 Validation

Validation was performed for a representative set of compounds (QC sample, *n* = 56) taking into account recent recommendations for the validation of qualitative HRMS screening [27]. The validation experiments included specificity, limit of identification (LOI), stability in pre‐treated samples, intra‐ and inter‐day repeatability, and matrix effects. Specificity was assessed by analyzing 10 drug‐free urine samples. The LOI was determined in six different blank urine samples by a dilution series starting at the QC concentration. Stability of compounds at QC concentration in pre‐treated samples was investigated by analyzing one batch of samples after pretreatment and again after three and five days of storage in the autosampler (10 °C). The repeatability for the 56 compounds was investigated at QC concentration by analyzing in parallel six different blank urine samples in one batch (intra‐assay, *n* = 6) and in two separate batches (inter‐assay, *n* = 12). Matrix effect was determined in a manner similar to other DS approaches for HRMS screening [28, 29, 30]. The studied compounds were spiked both in water (*n* = 3) and in blank urine (*n* = 6) at the QC concentration. The precursor peak heights in the matrix were compared with the peak heights of compounds prepared in water.

In the final stage of validation, authentic urine samples (*n* = 192) were analyzed, and the results were compared with those acquired earlier using SPE and conventional ESI (SPE‐ESI). The SPE‐ESI method, described in detail elsewhere [4], was the same as the current method except for sample preparation and ionization. In the SPE‐ESI method, urine samples were extracted with dual‐mode solid‐phase extraction (C4 + cation exchange) after hydrolysis with β‐glucuronidase.

## 3 Results and Discussion

A method for comprehensive toxicological drug screening in clinical urine samples by DS‐UHPLC‐VIP‐HESI‐QTOFMS was developed. Following a post‐hydrolysis dilution step, samples were chromatographed on a C18 column and introduced into the QTOFMS instrument applying the novel VIP‐HESI ion source in positive ion mode. The QTOFMS was set to continuously cycle between low collision energy MS and elevated collision energy (bbCID) MS/MS, acquiring accurate mass precursor and fragment ion data in a single analysis. Compound identification was performed against an in‐house database with 1263 compound entries (980 with retention time), using an automated post‐run reverse database search with preset criteria for the mass accuracy of precursor and qualifier ions, precursor ion peak height, and retention time. Precursor ion isotopic pattern match was used as an additional identification parameter. Validation was performed for 56 representative drugs, including of specificity, LOI, stability in pre‐treated samples, intra‐ and inter‐day repeatability, and matrix effects. In addition, authentic clinical urine samples (*n* = 192) were analyzed, and the results were compared with those obtained earlier with a method equal to the current method, except for the use of SPE for sample preparation and conventional ESI prior to QTOFMS analysis.

### 3.1 Method Development and Validation of Results

A hydrolysis step enabled the detection of unconjugated drugs and their metabolites. None of the 10 negative urine samples gave false positive results, and the method proved to be specific and selective for all the compounds studied, excluding alpha‐PHP. The co‐eluting positional isomer alpha‐PHiP cannot be separated from alpha‐PHP, since they have similar RT and identical fragmentation pattern. All other isomeric compounds tested could be separated by retention time and/or compound specific fragmentation patterns.

Validation results determined for the 56 compounds are presented in Table 1. The LOI was considered the concentration at which the compounds were identified in all six urine samples by automated data processing with the preset identification criteria. The LOI ranged from 1 to 45 ng/mL, with a median of 5 ng/mL. Since sample preparation is a simple dilution, analyte loss is minimal and, consequently, recovery was not of interest. According to Helfer et al. [31], recovery using a DS approach reached almost 100% for most compounds.

Stability was estimated as the percentage of change in the precursor peak height between the first analysis and analysis after three and five days of storage in the temperature‐controlled autosampler. The stability of analytes for all the compounds studied was acceptable. After three days, the peak height of the precursor ion did not change significantly (*data not shown*). Even after five days of storage, the change in peak height was still acceptable for most of the compounds. Thus, a reanalysis of the sample even after having been kept in the autosampler for five days is possible. Moreover, these results allow for a long sample sequence over a weekend.

Both the intra‐ and inter‐assay repeatability of RT was at an acceptable level (< 1%) for most compounds. However, for some early eluting compounds, the repeatability of RT was poorer. In particular, the compounds co‐eluting with endogenous compounds, such as creatine or phenylacetylglutamine, showed some RT shift. RT shift was most pronounced for amphetamine and naloxone, and in urine samples with high specific gravity. Even though the repeatability of RT was greater than 1% for some compounds, the RT tolerance for identification (±0.30 min) was still enough for each compound. Deventer et al. [32] stated that RT shifts were more frequent in samples with specific gravity higher than 1.030 g/mL, with acidic compounds with reduced retention, basic compounds with increased retention, or analytes of interest eluting with high‐concentration analytes.

Mass accuracy of precursor ions was always better than 1 mDa, with the median intra‐ and inter‐assay mass accuracy being 0.12 mDa and 0.08 mDa, respectively. Intra‐ and inter‐assay mass accuracy of qualifier ions was always better than 2.10 mDa (*data not shown*). The fragment ions for bupropion (*m/z* 131.0730) and risperidone (*m/z* 191.1190) showed the greatest, yet still acceptable, mass errors of 2.10 mDa and 1.17 mDa, respectively, but mass accuracy for all the other qualifier ions was better than 1 mDa.

For the determination of matrix effect, the peak heights of compounds prepared in urine and in water were compared. Values below 100% indicated ion suppression and values above 100% indicated ion enhancement. For most compounds, the matrix effect was between 70 and 130%. Morphine and naloxone suffered from ion suppression caused by co‐elution with early eluting endogenous interferences, such as creatinine. Urine also contains salts, which tend to elute at the void volume of the reversed‐phase column, thus affecting the early‐eluting compounds [32]. Ion enhancement was observed particularly with buprenorphine, levomepromazine, methylphenidate, and risperidone. In addition to ion enhancement, the incomplete solubility of a compound in a water sample and the instability of a compound in water may also have affected the results. As expected, matrix does have an effect in a DS drug screening approach, in which a sample cleanup step is omitted. However, since the repeatability of mass accuracy and RT between different urine matrices were acceptable and the LOIs were sufficiently low, even these fairly high matrix effects could be accepted, as similarly concluded by Pope et al. [29]

### 3.2 Comparison With SPE‐ESI Using Authentic Urine Samples

A total of 192 authentic clinical urine samples were analyzed using the current method, and the results were compared with those obtained earlier with a method equal to the current method except for the use of SPE for sample preparation and conventional ESI prior to QTOFMS analysis. The samples were analyzed in eight batches that each included 24 samples and a control sample. The results were evaluated in terms of number of identified compounds, comparison of signal intensities, and number of false negatives and positives. In this comparison, the total number of compounds identified was 1017 and, in general, detections were similar with both methods. The major differences were related to specific compounds, for which recovery has been known to be low with SPE, namely pregabalin, gabapentin, and ritalinic acid. In the preliminary tests preceding this study, we analyzed identical SPE extracts by both ionization techniques. To achieve comparable intensities between the techniques, we decreased the injection volume for the more sensitive VIP‐HESI and still found systematically higher intensities with this technique for amphetamines, benzodiazepines, gabapentinoids, and ritalinic acid, while other compounds showed similar or slightly higher intensities.

Between the two methods, 13 differing results were obtained. In six cases, the current method had not detected the excessively saturated pregabalin or gabapentin peaks that were outside the RT window (±0.30 min). The peaks were, however, easily identified via visual examination of the base peak chromatogram. In the remaining seven cases, small benzodiazepine peaks with concentrations close to the LOI were identified by one of the methods, but not by the other. These discrepancies could be related to benzodiazepine transformations during storage.

With the current method, two positive results were obtained for gabapentin, which were negative with SPE‐ESI. As recovery for gabapentinoids is poor with SPE‐ESI, it is not possible to evaluate the veracity of these observations. These were relatively small peaks with signal intensities below 100,000 counts, whereas for typical gabapentinoid peaks the signal intensities are usually several million counts.

**FIGURE 1:**
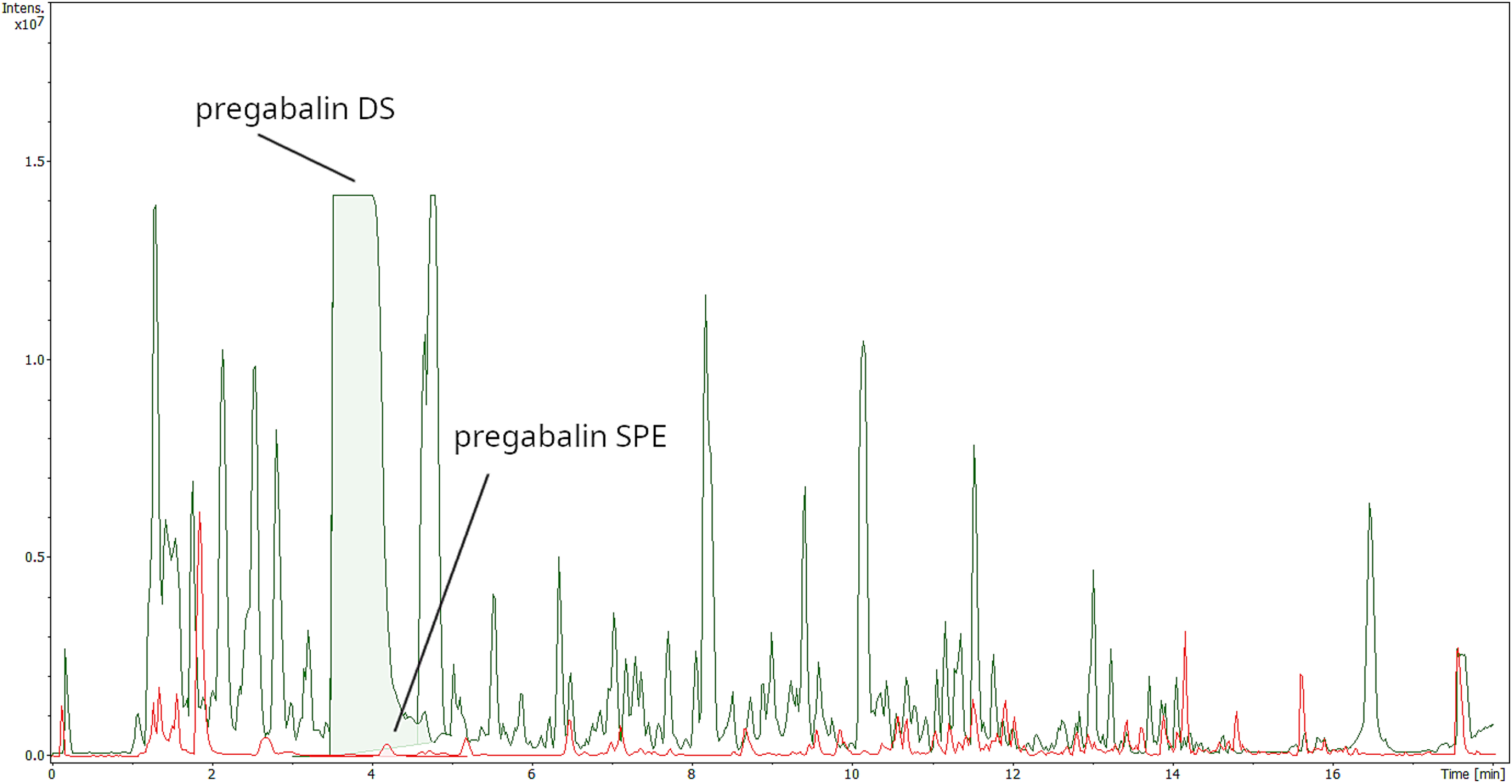
Base peak chromatograms (BPC) after dilute‐and‐shoot (DS, green) and solid phase (SPE, red) sample preparation, with DS showing a markedly enhanced intensity level, especially for labelled pregabalin. Additional findings are buprenorphine, norbuprenorphine, naloxone, tetrahydrocannabinolic acid, and lorazepam, all in low concentration.

As urine gabapentinoid concentrations are typically from tens to hundreds of mg/L [33, 34], high signal intensities can be expected (Figure 1). The false negative gabapentinoid results were due to RT shifts rather than poor mass accuracy due to detector saturation, indicating the excellent dynamic range of the instrument. We overcame this issue by increasing the RT tolerance of gabapentinoids. However, there remains a risk of false negative results for an analyte due to ion suppression caused by a co‐eluting gabapentinoid. Amphetamine (4.53 min) elutes close to pregabalin (4.18 min) and gabapentin (4.35 min), and such co‐elution may cause ion suppression for the precursor ion of amphetamine in the low collision‐energy data, but not for the amphetamine fragment ions (*m/z* 91.0542 and *m/z* 119.0855) acquired from the high collision‐energy data. Nonetheless, routine case work has demonstrated that this kind of precursor ion suppression only occurred in 0.3% of the samples during a period of two months before writing this article, and these cases were subsequently analyzed using a separate targeted method.

For ritalinic acid, the increase in signal intensity using the current method varied greatly, but was in general more than ten‐fold. Additional systematic increase in signal intensity level was observed for amphetamine, methamphetamine, and benzoylecgonine. Systematic decrease in signal level was observed for mirtazapine, carboxy‐tetrahydrocannabinol (THC‐COOH), and morphine, but without affecting their detectability. The overall median signal level in all positive findings was 38% higher with the current method than with SPE‐ESI.

The time required to process the data or interpret the results did not depend on sample preparation or ionization technology.

### 3.3 Method Robustness

During a 7.5‐month observation period, more than 7500 DS injections were performed on a single analytical column using the current method. Instrument downtime due to malfunctions was low due to the robustness of the instrument and well‐planned operations for preventive maintenance. As the sample preparation procedure involved post‐hydrolysis dilution and centrifugation, the amount of solubilized enzyme was reduced in the sample, increasing the lifespan of the column. In previously published DS studies, the column lifespan was shorter: Chindarkar et al. [28] reached 300–400 injections per column, Pope et al. [29] on average 2550 injections per column, and Saleh et al. [35] used the column for over 3000 injections.

In the current method, the RT of compounds in the system‐suitability test (amitriptyline, atenolol, dibenzepin, oxazepam, and warfarin) analyzed at the beginning of each sample sequence remained stable (RSD% below 1%) during the lifespan of a single analytical column (HSS T3, 150 × 2.1 mm, 1.8 μm). Pope et al. [29] used a similar column from the same vendor (HSS C18, 150 × 2.1 mm, 1.8 μm) and showed the stability between 13 columns to be acceptable, with only minor variation in retention times. Other quality assurance routines included a QC‐sample analyzed within each sample batch, stability monitoring (intensity, mass accuracy, RT) of QC‐compounds using a control chart, and verifying the solvent quality with a blank injection. In the external instrument calibration performed before each sample sequence, the intensity and mass resolving power for the calibrant ions (*m/z* 158.9641, 226.9515, and 362.9263) remained at the expected level confirming the stability of instrument operation.

The current method has been in routine use in our laboratory for about a year at the time of writing this manuscript. Sample sequences lasting two or three days included wash cycles within the sequence and a 50‐min gradient wash after each sequence. In addition, the ion source was cleaned after each sequence with more extensive decontamination at least once a month. Despite these cleaning steps, the precolumn had to be changed weekly on average, whereas with the former SPE approach it was changed approximately every three months.

### 3.4 Comparison With Previous Studies

Table 2 summarizes the most relevant published HRMS studies for drugs and drugs of abuse in forensic toxicology using the DS approach [26, 28, 29, 30, 31, 35, 36, 39, 43]. In addition, several papers have been published using DS for the screening of performance‐enhancing drugs prohibited in sport [38, 40, 41, 42, 44, 45, 46], although those doping control studies involving equine urine [45], a separation method other than reversed phase liquid chromatography [46], or a method comprising only anabolic steroids [44] were excluded from Table 2. Of the studies listed in Table 2, the matrix was post‐mortem urine in two cases [26, 36], whereas the other studies concerned urine samples from living persons.

**TABLE 2:** DS‐HRMS studies for the screening of drugs and drugs of abuse in urine samples for forensic and sports drug testing.

Reference	Dilution	Hydrolysis (y/n)	HRMS instrument	Ionization (+/−)	Data acquisition	Coverage of library	LOI (ng/mL)
[36]	1:9	n	Thermo Q Exactive Orbitrap	+	DIA	Tox Explorer™ library	Not known
[31]	1:3, 1:10	n	Thermo Q Exactive Orbitrap	+/−	DDA	> 3200 [37]	10–1000 ng/mL (1:3)
[29]	1:5	n	Waters QTOFMS	+, −	DIA	1400 (in‐house+Waters UNIFI)	2–100, mostly < 25
[26]	1:10	y	ABSciex QTOFMS	+, −	DDA	480 (RT for 365)	< 10 (for 90% of studied analytes)
[38], D	9:10^a^	n	Thermo Q Exactive Orbitrap	+, −	DIA	203	0.1–25 (LOD)
[39]	1:5	n	Thermo Q Exactive Orbitrap	+	DIA, PRM	148	0.5–50
[40], D	1:10	n	Thermo Exactive Orbitrap	+, −	DIA	122	5–500
[41], D	1:1	n	Waters QTOFMS	+, −	DIA	103	1–500
[42], D	1:50	n	Thermo Q Exactive Orbitrap	+	DIA	81	0.005–5
[30]	1:5	n	Agilent QTOFMS	+, −	DIA	62	2.8–187.5 (S/N = 3)
[28]	1:5	y	Waters QTOFMS	+, −	DIA	61	100 for most drugs
[43]	1:10	y	Thermo Exactive Orbitrap	+	DIA	29	2–100 (cutoff, no LOI determined in the study)
[35]	1:5	n	Waters QTOFMS	+	DIA	9	0.85–9.4 (LOD, S/N = 3), 2.8–41 (LOQ, S/N = 10)

Abbreviations: D, doping; DDA, data‐dependent acquisition; DIA, data‐independent acquisition; HRMS, high‐resolution mass spectrometry; LOD, limit of detection; LOI, limit of identification; LOQ, limit of quantification; PRM, parallel reaction monitoring; S/N, signal‐to‐noise ratio.

^a^ Only 10 μL internal standard added, positive (+)/negative (−) ionization.

Some of the DS approaches in earlier studies have included enzymatic hydrolysis with β‐glucuronidase [13, 17, 22, 23, 28, 43] or β‐glucuronidase/arylsulfatase [26]. Adding an enzyme for hydrolysis increases the amount of matrix in the sample [28], but post‐hydrolysis centrifugation removes most of the weighty enzyme after the incubation [17, 23, 26, 28]. A few studies applied a post‐hydrolysis dilution to further dilute the sample prior to analysis [13, 17, 26].

The most common data acquisition mode used with HRMS was data‐independent acquisition with both positive and negative ionization. Most studies also collected fragment data [26, 28, 29, 31, 36, 38, 42]. Two studies used data‐dependent acquisition, where the full scan served as a survey scan prior to high energy CID [26, 31]. In three studies, the method did not include high energy CID although QTOFMS was accessible [30, 35, 41]. However, in‐source fragments and adduct‐ions were used as secondary diagnostic ions for screening [41]. To acquire fragment data, a separate MS/MS function with compound‐specific collision energies was applied [30, 41]. Since fragmentation data were not available to Giron et al. [40], they used additional ions, such as adducts, isotopes (for compounds containing Br, Cl), and fragments formed from water cleavage from the protonated molecule, as a second diagnostic ion to increase identification power.

The LOIs of our current method were similar to or better than those reported in the other studies using DS and HRMS [26, 28, 29, 30, 31, 35]. In the current method, the early eluting morphine had the highest LOI of all (45 ng/mL), due to ion suppression caused by the co‐eluting endogenous biomolecule, creatinine. However, this level was still acceptable for screening purposes and lower than in most of the other studies cited. Indeed, many studies reported significant ion suppression for early‐eluting opiates [21, 23, 28, 29, 47]. The autosampler stabilities presented here were better than reported by Pope et al. [29], who concluded that the autosampler stability in their study was generally acceptable, but in practice re‐extracted samples were analyzed if a re‐analysis was necessary. In three previous methods [29, 31, 36], the scope of analysis was larger than in the current method, whereas in this respect the current method outperformed those methods applying a hydrolysis step [26, 28, 43].

## 4 Conclusions

We have described an improved way to perform urine drug screening by LC‐QTOFMS. The DS approach reduced manual workload, minimized the time and costs of sample preparation, and increased recoveries for drugs with challenging extraction characteristics as compared to an SPE approach. The recent advancements in QTOFMS technology, addressing the VIP‐HESI ion source, enabled DS to be applied to complex urine matrices. Non‐targeted data acquisition together with an automated post‐targeted database search provided immediate qualitative results for hundreds of drugs, while offering the possibility of retrospective data mining in special cases. The maintenance involved with the current method was somewhat more laborious than that of a similar method based on SPE, but this issue was compensated for by the other advantages. The LOIs of the current method were the same or better than those of the previously published DS‐HRMS methods.

## Conflicts of Interest

The authors declare no conflicts of interest.

## Data Availability

Research data are not shared.
